# Transcriptomic and Metabolomic Analyses of the Piz-t-Mediated Resistance in Rice against *Magnaporthe oryzae*

**DOI:** 10.3390/plants13233408

**Published:** 2024-12-04

**Authors:** Naeyeoung Choi, Xiao Xu, Pengfei Bai, Yanfang Liu, Shaoxing Dai, Matthew Bernier, Yun Lin, Yuese Ning, Joshua J. Blakeslee, Guo-Liang Wang

**Affiliations:** 1 Department of Plant Pathology, The Ohio State University, Columbus, OH 43210, USA; choi.1606@buckeyemail.osu.edu (N.C.); pengfei.bai1988@gmail.com (P.B.); 2 State Key Laboratory for Biology of Plant Diseases and Insect Pests, Institute of Plant Protection, Chinese Academy of Agricultural Sciences, Beijing 100193, China; xuxiaoo9@163.com (X.X.); ningyuese@caas.cn (Y.N.); 3 Quality Standard and Testing Technology Research Institute, Yunnan Academy of Agricultural Sciences, Kunming 650200, China; liuyf528@163.com; 4 Institute of Primate Translational Medicine, Kunming University of Science and Technology, Kunming 650500, China; daisx@lpbr.cn; 5 Campus Chemical Instrumentation Center (CCIC), The Ohio State University, Columbus, OH 43210, USA; bernier.19@osu.edu; 6 Department of Horticulture and Crop Science, The Ohio State University, Columbus, OH 43210, USA; lin.1418@buckeyemail.osu.edu; 7 Laboratory for the Analysis of Metabolites from Plants (LAMP), The Ohio State University, Columbus, OH 43210, USA

**Keywords:** rice blast, R gene-mediated resistance, metabolomics, transcriptomics

## Abstract

*Magnaporthe oryzae* causes devastating rice blast disease, significantly impacting rice production in many countries. Among the many known resistance (R) genes, *Piz-t* confers broad-spectrum resistance to *M. oryzae* isolates and encodes a nucleotide-binding site leucine-rich repeat receptor (NLR). Although Piz-t-interacting proteins and those in the signal transduction pathway have been identified over the last decade, the Piz-t-mediated resistance has not been fully understood at the transcriptomic and metabolomic levels. In this study, we performed transcriptomic and metabolomic analyses in the *Piz-t* plants after inoculation with *M. oryzae.* The transcriptomic analysis identified a total of 15,571 differentially expressed genes (DEGs) from infected *Piz-t* and wild-type plants, with 2791 being *Piz-t-*specific. K-means clustering, GO term analysis, and KEGG enrichment pathway analyses of the total DEGs identified five groups of DEGs with distinct gene expression patterns at different time points post inoculation. GO term analysis of the 2791 *Piz-t*-specific DEGs revealed that pathways related to DNA organization, gene expression regulation, and cell division were highly enriched in the group, especially at early infection stages. The gene expression patterns in the transcriptomic datasets were well correlated with the metabolomic profiling. Broad-spectrum “pathway-level” metabolomic analyses indicated that terpenoid, phenylpropanoid, flavonoid, fatty acid, amino acid, glycolysis/TCA, and phenylalanine pathways were altered in the *Piz-t* plants after *M. oryzae* infection. This study offers new insights into the molecular dynamics of transcripts and metabolites in R-gene-mediated resistance against *M. oryzae* and provides candidates for enhancing rice blast resistance through the engineering of metabolic pathways.

## 1. Introduction

Rice (*Oryza sativa* L.) is a staple crop for about 50% of the global population, especially in Asia. Many pathogens infect rice and cause huge yield losses in rice production. Among them, the hemibiotrophic fungus *Magnaporthe oryzae*, syn. *Magnaporthe grisea*, is the causal agent of the destructive rice blast disease that occurs in 85 countries [1]. Although current cultural strategies such as reducing the use of nitrogen fertilizers, crop rotation, maintaining proper flood levels and applying fungicides, are employed, genetic resistance remains a widely used, economical, and effective strategy for controlling the disease in many countries. Importantly, members of the *M. oryzae* complex can also infect other cereals such as wheat, barley and corn, posing a threat to global food security [2].

The rice-*M. oryzae* pathosystem has emerged as an important model for studying plant–microbe interactions. To date, over 38 resistance (R) genes in rice have been cloned [3,4]. The majority of the cloned R genes encode a nucleotide-binding site leucine-rich repeat receptor (NLR), while a few others encode proteins such as a proline-rich metal binding protein, a B-lectin receptor kinase, receptor-like kinases and an ARM repeat protein. Regarding the cognate avirulence (*Avr*) genes of the cloned R genes, 14 *Avr* genes have been identified in *M. oryzae* with no sequence homology among them [3,4]. Following the gene-for-gene hypothesis, the recognition between most of the R genes in rice and their *Avr* genes in *M. oryzae* directly or indirectly triggers a hyposensitive reaction (HR), initiating a cascade of defense signaling events and robust downstream defense responses, such as significant transcriptomic and metabolomic changes in rice plants after *M. oryzae* infection [5,6]. RNA-seq has been used to analyze differential expression patterns of genes in pathogen-infected plants, and numerous differentially expressed genes (DEGs) have been identified that are associated with resistance or susceptibility [7]. For example, following *M. oryzae* infection, many DEGs were identified in rice that encode pattern recognition receptors (PRRs), enzymes for the generation of reactive oxygen species (ROS), pathogenesis-related (PR) proteins, and secondary metabolites [8,9,10,11]. Transcriptomic analyses therefore offer a glimpse into understanding these coordinated mechanisms of rice immune responses against *M. oryzae.*

Pathogen invasion triggers defense responses, such as cell wall modifications and synthesis of various metabolites with antimicrobial and antifungal activities [12,13]. Metabolomic profiling can identify specific synthesis and defense pathways altered in pathogen-treated plants vs. non-infected controls. Metabolomic analyses can be qualitative (identifying compounds found in infected vs. non-infected populations), quantitative (quantifying shifts in metabolites following infection or comparing relative accumulations in infected vs. non-infected populations), or a combination of both [14]. A recent study indicated that several metabolic pathways, including the phenylpropanoid, lignin, terpenoid and flavonoid biosynthesis pathways showed increased flux and accumulation of metabolites in three maize lesion mimics [15]. Another recent metabolomic study reported that different defense-related enzyme activities were found between resistant and susceptible cultivars of rice after *M. oryzae* infection [5]. In these experiments, it was found that the most enriched differentially accumulated metabolites were lipids and lipid-like molecules; phenylpropanoids and polyketides; organoheterocyclic compounds; organic acids and derivatives; and lignans, neolignans, and related compounds [5].

An integrated transcriptomic and metabolomic approach allows for the simultaneous analysis of transcripts and metabolites, providing a comprehensive view of transcriptome and metabolic changes after pathogen infection. A recent study showed that inoculation of *Xanthomonas oryzae* pv. *oryzae* in rice causes a rapid expression of many DEGs related to defense, as well as to the synthesis of various secondary metabolites [16]. Another integrated study revealed that potassium levels are important in determining rice susceptibility against *Sarocladium oryzae*, influencing the regulation of gene expressions and secondary metabolites [17]. Regarding *M. oryzae*, one study found that Moringa–chitosan nanoparticles suppress *M. oryzae* growth directly via multiple mechanisms related to alteration of gene expressions and multiple metabolomic pathways [18]. Although integrated analyses have been widely used for different pathosystems, there is limited information available on how genes and metabolites are regulated in R gene-mediated resistance, or how regulation of gene expression and metabolic pathways is integrated across multiple time points post infection.

The rice blast R gene *Piz-t* encodes an NLR and recognizes the product of the cognate *AvrPiz-t* gene in *M. oryzae* [19,20]. Previous studies have shown that AvrPiz-t does not interact with Piz-t directly, but instead binds to several AvrPiz-t interacting proteins (APIPs). For instance, the E3 ligases APIP6 and APIP10 ubiquitinate AvrPiz-t, resulting in the degradation of the latter; and, in return, AvrPiz-t ubiquitinates and degrades both APIP proteins [21,22]. Notably, although APIP10 does not interact with Piz-t, knocking down of *APIP10* causes Piz-t protein accumulation and increased cell death. Two studies showed that APIP5 not only interacts with and stabilizes Piz-t, but that APIP5 also functions as a transcription factor and an RNA-binding protein modulating cell death and immunity in rice [23,24]. Further, a recent study reported that two VOZ transcription factors link APIP10 and Piz-t to modulate immunity in rice [25]. While these studies have revealed important information regarding the signaling mechanisms underlying *Piz-t*-mediated resistance, it is not fully understood how the AvrPiz-t–Piz-t interaction causes transcriptomic and metabolomic reprograming in *Piz-t* plants following infection with an *AvrPiz-t-*bearing *M. oryzae* isolate.

In this study, we applied both transcriptomic and metabolomic profiling approaches to identify metabolic pathways which were either up- or down-regulated in the *M. oryzae-*resistant Nipponbare-Piz-t (NPB-Piz-t) transgenic line compared to the susceptible cultivar Nipponbare (NPB) following inoculation with the *AvrPiz-t*-containing *M. oryzae* isolate RO1-1. K-means clustering showed distinctive patterns of increased or decreased gene clusters in NPB-Piz-t compared to NPB across different time points post inoculation. Gene Ontology (GO) and Kyoto Encyclopedia of Genes and Genomes (KEGG) pathway analyses revealed that several groups of genes related to specific metabolomic pathways were highly expressed at both 96 and 120 hours post inoculation (hpi). Integrated transcriptomic and metabolomic analyses indicated that NPB-Piz-t exhibited increased synthesis and/or accumulation of multiple metabolic classes, including fatty acids, flavanols, and amino acids (particularly phenylalanine), with previously demonstrated anti-microbial activities. Our data provide new insights into the dynamics and interactions between changes in transcriptomic expression and metabolic shifts in *R*-gene-mediated resistance against *M. oryzae* and allow the identification of both metabolic pathways which may be used to enhance rice blast resistance through a combination of breeding and metabolic engineering approaches.

## 2. Results

### 2.1. Transcriptomic Profiling of NPB-Piz-t at Different Time Points After Inoculation

**Figure 1:**
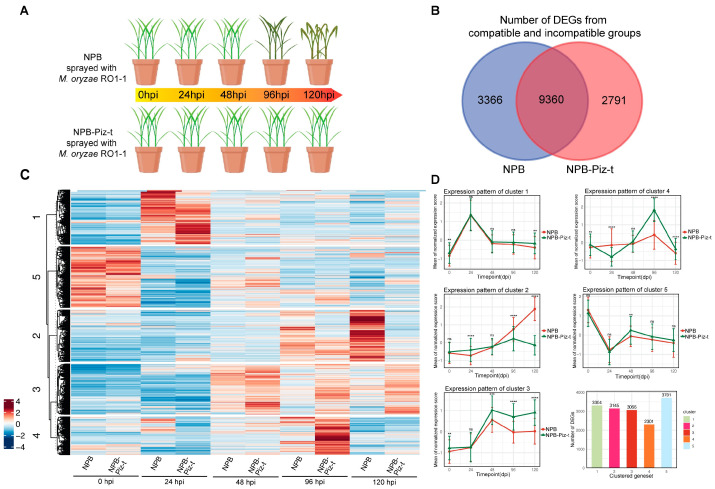
Transcriptome profiling of the *Piz-t*-mediated resistance. (**A**) Rice plants (NPB and NPB-Piz-t) were sprayed with *Magnaporthe oryzae* strain RO1-1. Sampling was collected at 0, 24, 48, 96 and 120 h post inoculation (hpi). (**B**) Venn diagram showing the combined number of differentially expressed genes (DEGs) identified in NPB and NPB-Piz-t groups at all time points. The 0 hpi time point from each group was used as the reference for normalization. (**C**) Heatmap displaying the expression profiles of DEGs in both NPB and NPB-Piz-t groups across the different time points. The color scale from blue to red represents low- to high-expression levels. The hierarchical clustering reveals distinct gene expression patterns in response to *M. oryzae* infection. (**D**) Line graphs illustrating the expression patterns of representative gene clusters from the heatmap. Each panel represents the mean expression levels of a specific gene set (clusters 01 to 05) across the sampled time points. Error bars indicate the standard error of the mean (SEM). Significant differences between the groups are marked with asterisks (** *p* < 0.01, *** *p* < 0.001, **** *p* < 0.0001).

Total mRNA was extracted from leaves of the NPB-Piz-t and NPB plants at 0, 24, 48, 96 and 120 hpi following inoculation with *M. oryzae* isolate RO1-1, which is incompatible with NPB-Piz-t and compatible with NPB. Zero hpi was used as a reference time point to analyze DEGs in NPB-Piz-t and NPB plants (Figure 1A). Transcriptomic analysis identified 15,571 DEGs exhibiting a minimum of a 2-fold change in expression and an adjusted *p*-value of less than 0.05 across all five time points post infection (Table S1). Among these, 2791 genes were either up-regulated or down-regulated specifically in NPB-Piz-t, while 3366 genes were up-or down-regulated specifically in NPB (Figure 1B, Table S1-1). Interestingly, 9360 DEGs were either induced or suppressed in a similar fashion in both NPB-Piz-t and NPB post infection (Figure 1B, Table S1-1). Before inoculation (0 hpi), only a small number of DEGs were found to be up- or down-regulated specifically in NPB-Piz-t (321 and 98, respectively). However, the number of up- or down-regulated DEGs unique to NPB-Piz-t increased over 10-fold by 24 hpi (4191 up-regulated and 4486 down-regulated), at 48 hpi (4460 up-regulated and 1208 down-regulated), at 96 hpi (5458 up-regulated and 1550 down-regulated), and at 120 hpi (4208 up-regulated and 2369 down-regulated) (Tables S1-2–S11).

All the identified DEGs were further analyzed using heatmap and K-means clustering analyses (Figure 1C,D, Table S2). The heatmap results indicated that the DEGs could be divided into five major groups based on their expression patterns across the five time points. At 0 hpi, both NPB-Piz-t and NPB showed similar expression patterns among the DEGs, but differences in DEG expression between the two lines began to appear after 24 hpi (Figure 1C). K-mean analysis also clustered the DEGs into five groups (Figure 1D). Clusters 1 and 5 did not exhibit significant differences between NPB-Piz-t and NPB. Instead, both clusters displayed synchronous up- and down-regulations in the two lines at the early infection stages, suggesting these genes might be involved in basal defense responses to *M. oryzae.* Notably, the DEGs in clusters 2, 3 and 4 (3145, 3066 and 2301, respectively) showed significant differences after infection, particularly after 48 hpi. These results suggest that *Piz-t*-mediated resistance causes significant differences in gene expression, starting at the early infection stages (24 hpi), and that more significant divergences in gene expression between NPB-Piz-t and NPB occur at 48, 96 and 120 hpi between the resistant and susceptible lines after RO1-1 inoculation.

### 2.2. GO Analysis of the DEGs in Three Clusters

**Figure 2:**
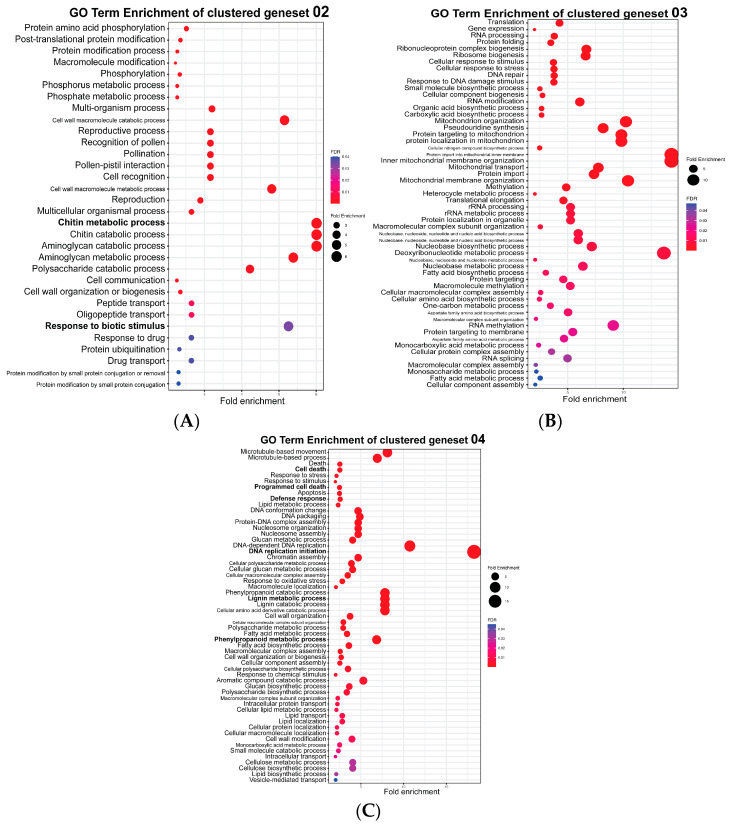
GO term analysis of gene clusters identified in the transcriptome data. A-C GO enrichment analysis for the clustered gene sets from cluster 2, cluster 3 and cluster 4 in Figure 1D, highlighting significantly enriched biological processes. Each panel shows the top enriched GO terms for a specific gene cluster, with fold enrichment >= 2.0 and false discovery rate (FDR) < 0.05. The size of the circles represents the fold enrichment, and the color indicates the FDR. (**A**). GO term enrichment of cluster 2. (**B**). GO term enrichment of cluster 3. (**C**). GO term enrichment of cluster 4.

Next, we performed GO analyses on the DEGs in clusters 2, 3 and 4. Overall, the DEGs in cluster 2 showed a relatively lower expression in NPB-Piz-t compared to NPB (Figure 1D, middle left, Table S2). Specifically, the expression of cluster 2 DEGs in NPB was slightly lower at 24 hpi but rapidly increased from 48 to 120 hpi. In contrast to this, the expression of these DEGs in NPB-Piz-t gradually increased from 24 to 96 hpi, after which expression was reduced slightly by 120 hpi. Analyses of these DEGs identified several metabolic pathways showing increased expression (>5 fold) of genes in these pathways. Metabolic pathways linked to increased DEG expression included chitin metabolic processes, chitin catabolism, aminoglycan catabolic processes, aminoglycan metabolic processes, cell wall macromolecule metabolic processes, and responses to biotic stimuli (Figure 2A) Notably, 300 genes involved in chitin catabolic processes, and 66 genes involved in biotic stimulus responses were enriched in this cluster of DEGs (Figure 2A, Table S2), suggesting the import ance of these two biological processes in resistance to *M. oryzae*.

In general, the expression of DEGs in cluster 3 was higher in NPB-Piz-t compared to NPB, especially at 48, 96 and 120 hpi. While at 0 and 24 hpi, the expression levels of cluster 3 DEGs were similar between NPB-Piz-t and NPB, by 48 to 120 hpi, the expression levels of cluster 3 DEGs in NPB-Piz-t were much higher than in NPB (Figure 1D, lower left). GO term analysis revealed that seven biological processes were highly enriched (≥10-fold) in this cluster of DEGs in NPB-Piz-t, including processes of protein import into the mitochondrial inner membrane, organization of the inner mitochondrial membrane, deoxyribonucleotide metabolism, organization of the mitochondrial membrane, organization of the mitochondria, protein targeting of the mitochondria, and protein translocation into the mitochondria (Figure 2B). These results suggest that mitochondria might play important roles in resistance to *M. oryzae* [26].

The expression of the DEGs in cluster 4 had a different pattern of expression in NPB-Piz-t vs. NPB (Figure 1D, upper right). In NPB-Piz-t, these DEGs were down-regulated at 24 hpi, but this trend reversed at 24 hpi, and global expression of the DEGs was up-regulated by 96 hpi, followed by down-regulation at 120 hpi. Conversely, the expression of cluster 4 DEGs in NPB showed a small increase from 0 to 96 hpi, but was down-regulated at 120 hpi. GO term analysis revealed that these genes were linked to biological processes involved in DNA replication and initiation, as well as DNA-dependent DNA replication (DEGs involved in these two biological pathway clusters were enriched 15- and 10-fold, respectively, in NPB-Piz-t) (Figure 2C). Unsurprisingly, given the well-documented role of phenylpropanoids in plant–pathogen interactions [27], several DEGs linked to phenylpropanoid metabolism (particularly catabolism) and lignin metabolism (both synthesis and catabolism), were also significantly enriched (7–8 fold), along with DEGs associated with amino and carboxylic acid metabolism (Figure 2C).

### 2.3. KEGG Enrichment Analysis of the DEGs at Different Time Points

Based on K-means clustering and GO analyses, the transcriptomes of NPB-Piz-t and

**Figure 3:**
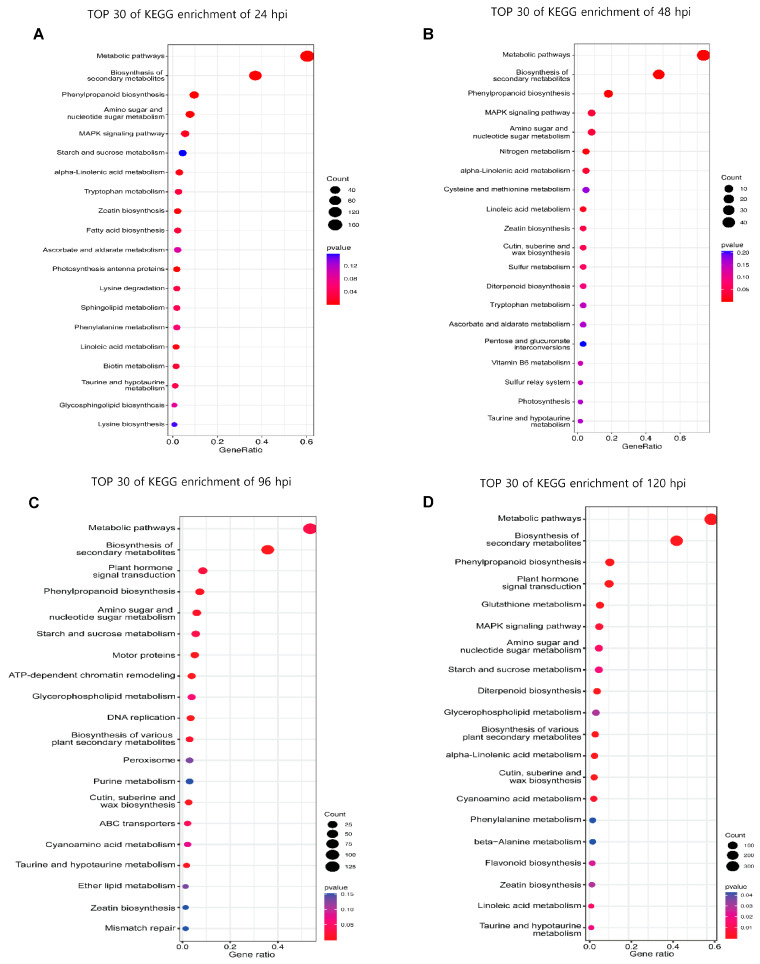
KEGG pathway analysis of the identified DEGs. A-D KEGG pathway enrichment analysis at different time points. The bar chart shows the gene ratio for each significantly enriched pathway, including metabolic pathways, biosynthesis of secondary metabolites, phenylpropanoid biosynthesis and plant hormone signal transduction. The size of the dots represents the gene count, and the color indicates the *p*-value, with red indicating higher significance. (**A**). KEGG enrichment of 24 hpi. (**B**). KEGG enrichment of 48 hpi. (**C**). KEGG enrichment of 96 hpi. (**D**). KEGG enrichment of 120 hpi.

NPB showed significant differences at 24, 48, 96 and 120 hpi. Because of this, the DEGs at all time points were subjected to KEGG enrichment analysis (Figure 3, Table S3). Interestingly, KEGG enrichment analysis of both datasets revealed that the same pathways were enriched across all time points. Among the top 30 enriched pathways in NPB-Piz-t, metabolic pathways were the most highly enriched at all time points (Figure 3A–D). Genes involved in the biosynthesis of secondary metabolites showed the greatest levels of enrichment, and were most represented in the 30 most highly enriched genes at all time points: 24 hpi (100 genes), 48 hpi (29 genes), 96 hpi (210 genes), and 120 hpi (573 genes) (Figure 3, Table S3). Of the up-regulated genes involved in metabolic pathways, 24 (all involved in sugar and amino acid biosynthesis) showed increased expression at both 96 and 120 hpi but not at 24 and 48 hpi (Table S4). Of the genes involved in secondary metabolism, those linked to phenylpropanoid metabolic pathways were significantly enriched in NPB-Piz-t at all time points: 24 hpi (26 genes), 48 hpi (11 genes) 96 hpi (17 genes) and 120 hpi (56 genes) (Figure 3, Table S4).

In addition to the pathways identified above, in which transcripts with increased expression were identified across four different time points, our KEGG enrichment analyses also identified DEGs enriched in NPB-Piz-t at each timepoint. For example, genes involved in sphingolipid metabolism and biotin metabolism were enriched in NPB-Piz-t only at 24 hpi (Figure 3A); genes involved in sulfur metabolism, pentose and glucuronate interconversions, and vitamin B6 metabolism were enriched in NPB-Piz-t at 48 hpi (Figure 3B); and genes involved in plant hormone transduction, starch metabolism, ATP-dependent chromatin remodeling, purine metabolism, and the expression of ABC transporters were enriched in NPB-Piz-t specifically at 96 hpi (Figure 3C). Lastly, genes involved in glutathione metabolism, diterpenoid biosynthesis, and linoleic metabolism were enriched in NPB-Piz-t only at 120 hpi (Figure 3D).

### 2.4. GO Enrichment Analysis of the 2791-Piz-t Specific DEGs

**Figure 4:**
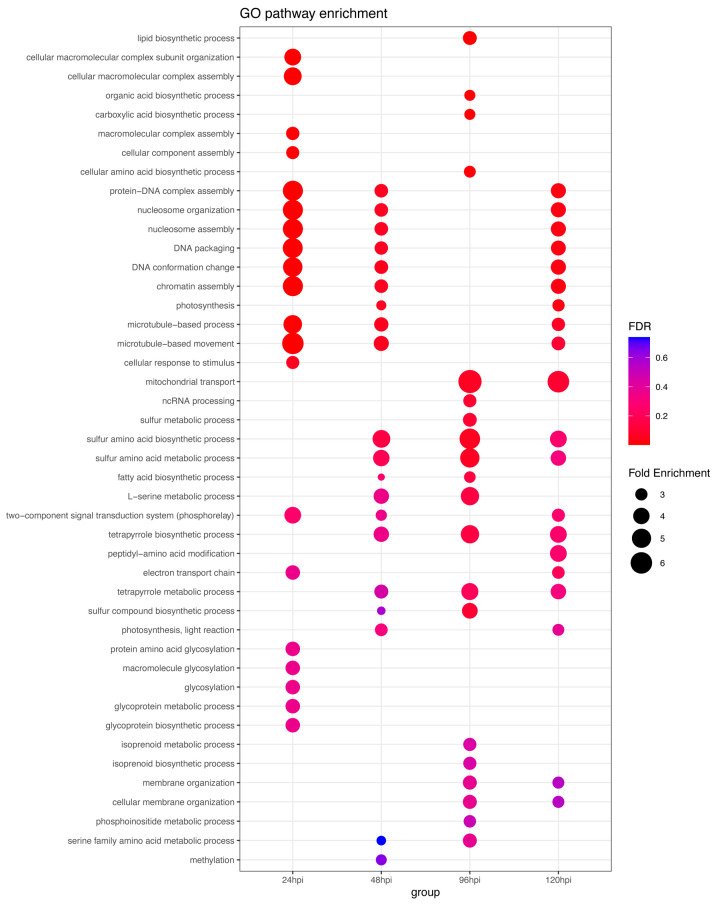
GO term analysis of 2791-*Piz-t* specific DEGs. Top 20 GO terms from each timepoint were plotted together. The size of the dots represents the gene count, and the color indicates the *p*-value, with red indicating higher significance functions related to DNA organization and gene expression regulation are important for *Piz-t*-mediated resistance.

We performed GO term analysis to determine the enrichment of biological functions at all time points after inoculation using the *Piz-t* specific 2791 DEGs (Figure 1B), and plotted the top 20 GO terms from each time point, in Figure 4. At 24 hpi, the protein DNA complex assembly (nine genes), nucleosome organization (nine genes), DNA packaging (nine genes), DNA conformation change (ten genes), chromatin assembly (nine genes) and microtubule-based movement (nine genes) were significantly enriched in NPB-Piz-t (Table S5). At 48 hpi, the L-serine metabolic process (five genes), the sulfur amino acid biosynthetic process (six genes) and the metabolomic process (six genes) were significantly enriched in NPB-Piz-t (Table S5). At 96 hpi, mitochondrial transport (five genes), the tetrapyrrole biosynthetic process (five genes), the sulfur amino acid biosynthetic process (six genes) and the metabolomic process (six genes) showed enrichment in NPB-Piz-t (Table S5). Lastly, at 120 hpi, the mitochondrial transport process (five genes) was the only highly enriched GO cluster in NPB-Piz-t (Table S5). Interestingly, our analyses indicated that biological processes involved in the DNA–protein complex assembly, nucleosome organization and assembly, DNA packaging, DNA conformation change, chromatin assembly, microtubule-based processes, and microtubule-based movement were significantly enriched across all time points, except 96 hpi. These results suggest that the cellular

### 2.5. Metabolomic Profiling of NPB-Piz-t at Different Time Points

In parallel with our KEGG enrichment and GO analyses, which revealed significant changes in the genes involved in several metabolomic pathways in NPB-Piz-t during blast infection, we also performed non-targeted metabolomic profiling of NPB-Piz-t vs. NPB following inoculation with blast strain RO1-1. In these studies, leaves of two-week-old seedlings were used for inoculation of RO1-1. After inoculation of RO1-1, the inoculated leaves were harvested at three different time points, 0, 48 and 96 hpi. Extracted metabolites were analyzed with liquid chromatography quadrupole time-of-flight mass spectrometry (LC-qTOF-MS) and feature identification was performed using a custom algorithm which screened individual features against the KEGG, Plant Metabolic Network (PMN), and Chemspider databases.

**Figure 5:**
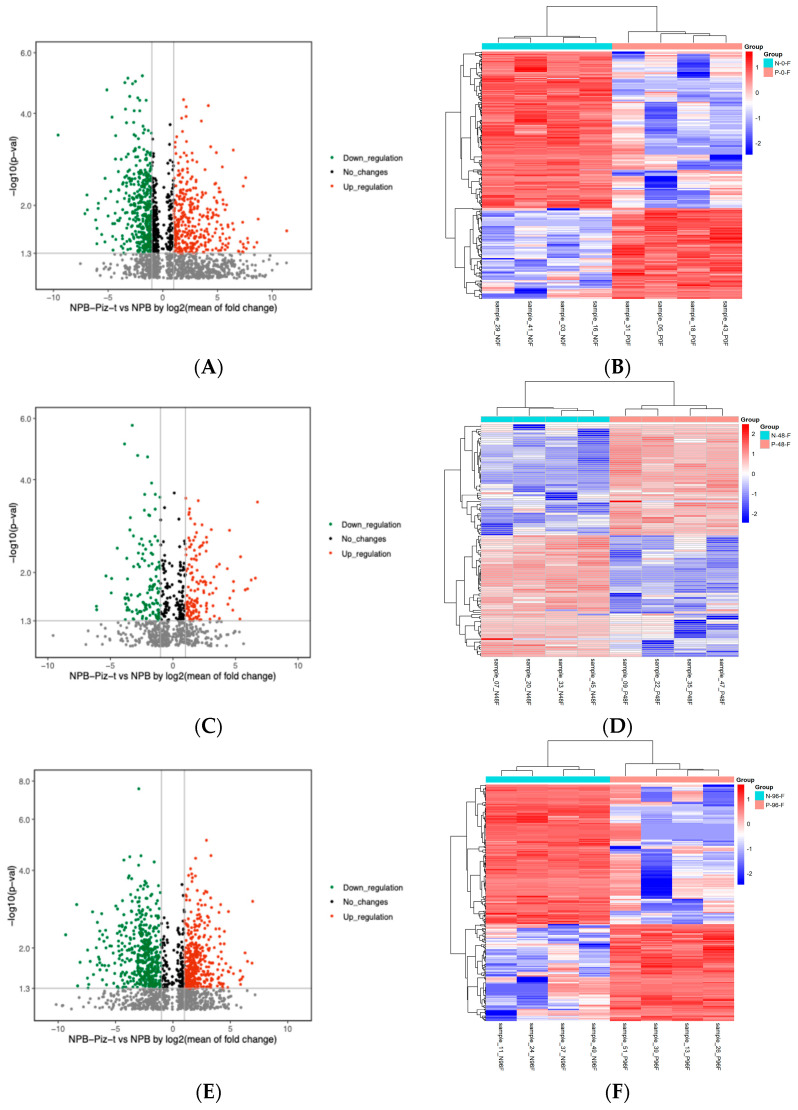
Metabolomic analysis of the *Piz-t-*mediated resistance after *M. oryzae* infection. (**A**,**C**,**E**). Volcano plot of the metabolites showing altered expression patterns in NPB-Piz-t in comparison with NPB. A total of 347, 137 and 423 metabolites showed increased expression at 0, 48 and 96 hpi after RO1-1 inoculation in NPB-Piz-t, while 441, 124 and 461 metabolites showed decreased expression patterns at 0, 48 and 96 hpi after RO1-1 inoculation in NPB-Piz-t. (**B**,**D**,**F**). Heatmaps of differentially accumulated metabolites in 0, 48 and 96 hpi after RO1-1 inoculation in NPB-Piz-t, with *p* values < 0.05.

Volcano plots of the metabolomic data revealed differences in the accumulation of metabolites between NPB-Piz-t and NPB across all three time points (Table S6). Interestingly, these two lines exhibited quantifiable shifts in metabolism even prior to infection, as NPB-Piz-t showed increased accumulation of 347 metabolites and decreased accumulation of 411 metabolites, even at 0 hpi (Figure 5A). By 48 hpi, these numbers had shifted, and there were only 137 metabolites showing increased accumulation and 124 metabolites showing decreased accumulation in NPB-Piz-t (Figure 5C). However, at 96 hpi, the number of those induced and suppressed specifically in NPB-Piz-t was similar to those at 0 hpi (423 and 461, respectively) (Figure 5E).

To confirm the volcano plot results, we analyzed the data using heatmap clustering (Figure 5B,D,F, Table S6). At 0 hpi, all four samples of NPB-Piz-t and NPB were clustered together (Figure 5B). At 48 hpi, the total of numbers of metabolites showing both increased and reduced levels of accumulation decreased in both lines compared to 0 hpi (Figure 5D). Additionally, the number of metabolites showing decreased accumulation was higher than the number of metabolites showing increased accumulation across this time course. At 96 hpi, the total numbers of metabolites showing both increased and decreased levels of accumulation returned to levels comparable to those at 0 hpi in both NPB-Piz-t and NPB (Figure 5F). These data suggest that the *M. oryzae* infection may suppress host metabolite biosynthesis in the *Piz-t* plants at the early-to-middle infection stages.

**Figure 6:**
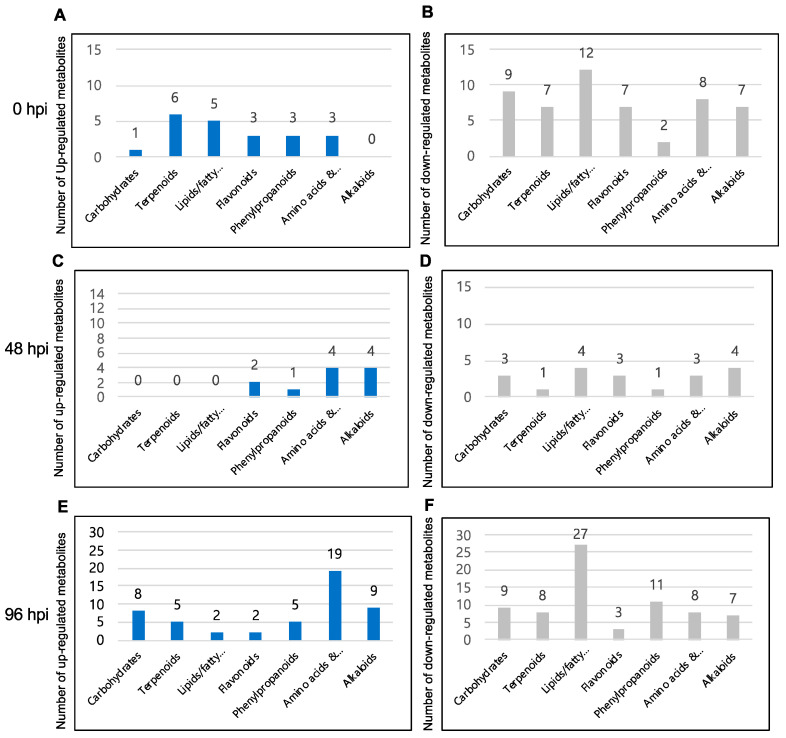
The number of metabolites in different biological pathways that were up- and down-regulated in *NPB-Piz-t* compared with NPB after inoculation. (**A**,**C**,**E**). The number of metabolites being up-regulated in *NPB-Piz-t* vs. NPB after inoculation of RO1-1. (**B**,**D**,**F**). The number of metabolites being down-regulated in *NPB-Piz-t* vs. NPB after inoculation of RO1-1.

To identify specific clusters of metabolites and metabolic pathways affected by blast infection across the three time points, we conducted tentative analyses of metabolite features using the KEGG, PMN, and Chemspider databases. When screening features, we selected the compounds showing the greatest shifts in accumulation, both upwards and downwards (up to 200 in each category), in NPB-Piz-t vs. NPB at the three time points post infection collected in this study (0, 48 and 96 hpi). Based on these data, we identified the metabolic pathways impacted most in NPB-Piz-t across 0, 48, and 96 time points as terpenoid metabolism, flavonoid metabolism, phenylpropanoid metabolism, lipid/fatty acid/membrane component metabolism, amino acid metabolism, carbohydrate metabolism, and alkaloid metabolism (Figure 6). Additional metabolic pathways identified as being up-regulated in NPB-Piz-t across multiple time-points included benzoic acid, propanoate, and biotin, metabolism, while additional pathways identified as being down-regulated in NPB-Piz-t throughout the course of infection included both propanoic and amino benzoic acid metabolism. Changes to specific metabolic pathways are discussed below:

Terpenoids: Terpenoids were a class of metabolites that showed significant differential accumulation between NPB-Piz-t and NPB across all time-points post infection. In particular, putatively identified monterpene-related compounds (especially those tentatively identified as loganin-related terpenoids) showed the greatest shifts in accumulation in NPB-Piz-t compared to control, exhibiting decreased accumulation at 0 and 48 hpi, but increased accumulation at 96 hpi. Greater numbers of terpenes showed decreased accumulation than increased accumulation in NPB-Piz-t at 0 hpi (six increased, seven decreased). At 48 hpi, the number of terpenes showing either increased or reduced accumulation decreased (none with increased accumulation, one with decreased accumulation). At 96 hpi, the number of putative terpenes showing both increased and decreased accumulation in NPB-Piz-t increased compared to 48 hpi (five increased and eight decreased accumulation). Most of the terpenes showing decreased accumulation were either potentially loganin-related compounds or unknowns; and the majority of terpenes showing increased accumulation were unknown/not-identified (Tables S7–S12). Interestingly, and somewhat unexpectedly, at 96 dpi, we observed decreased accumulation of a diterpenoid tentatively identified as the phytoalexin phytocassene D.

Phenylpropanoids and flavonoids: Phenylpropanoid and flavonoid metabolites also showed altered accumulation in NPB-Piz-t across all three time points (Figure 6). At 0 hpi, three putative phenylpropanoids showed increased accumulation, while two exhibited decreased accumulation. AT 48 hpi, the numbers of phenylpropanoids showing altered accumulation were decreased (one with increased accumulation, one with decreased accumulation). By 96 hpi, five phenylpropanoids exhibited increased accumulation, while twelve showed decreased accumulation. Notably, a compound identified as isocoumarin showed increased accumulation at 0 hpi. Isocoumarin has been studied as functional antimicrobial agent against *Pseudomonas syringae* [28]. At 0 hpi, flavonoids in NPB-Piz-t also showed altered accumulation, with three exhibiting increased accumulation and seven decreased accumulation. By 48 hpi, two potential flavonoids were showing increased accumulation and three decreased accumulations in *Piz-t* vs. WT. At 96 hpi, two flavonoids showed increased accumulation, while three exhibited decreased accumulation in NPB-Piz-t. Interestingly, a compound tentatively identified as glepidotin B showed increased accumulation at 0 hpi and a second, putatively identified as angusticornin B, accumulated in NPB-Piz-t at 48 hpi. Both of these flavonoid-related compounds have been hypothesized to function as antimicrobial agents [29,30]. Additional putative flavonoids showing increased accumulation in NPB-Piz-t throughout the course of infection included heliespirone B, (5Z)-7-Isopropyl-4,10-bis (methylene)-5-cyclodecen-1-ol and isorhamnetin. Contrastingly, the putative flavonoids eugenin, apixaban, 5,7-Dihydroxy-2-(4-hydroxyphenyl)-3-methoxy-4H-chromen-4-one (3-methyl-kaempferol) and plantagoside showed decreased accumulation in NPB-Piz-t vs. NPB across all time points (Tables S7–S12).

Fatty acids, sterols, and membrane-related compounds: Fatty acids were also identified as a key group of metabolites exhibiting shifts in accumulation in NPB-Piz-t during blast infection. Fatty acids are involved in several aspects of plant defense, and fatty acid metabolism has also been linked to the production and regulation of the accumulation of reactive oxygen species (ROS) [31]. At 0 hpi, five putative fatty acids/lipids/membrane components showed increased accumulation and twelve showed decreased accumulation in NPB-Piz-t vs. NPB (Figure 5). Notably, the membrane-related compound showing the greatest increase in accumulation 0 hpi was a feature putatively identified as the sphingolipid metabolite sphingosine 1-phosphate. It has been reported that sphingolipids play roles in programmed cell death (PCD) and hypersensitive responses (HRs) in plants [32]. Similar to trends observed for other metabolic clusters above, the number of fatty acids exhibiting both increased and decreased accumulation decreased at 48 hpi (none increased and four decreased). However, the number of fatty acids with decreased accumulation in NPB-Piz-t dramatically increased at 96 hpi (two increased and twenty-seven decreased). Interestingly, the biosynthesis of lipids putatively identified to be linked to cell membrane and energy storage, such as features tentatively identified as propanedioic acid and octadecanoic acid, appeared to be significantly decreased at 96 hpi (Tables S7–S12).

Amino acids and related metabolites: Several amino acids and metabolites related to amino acid metabolism were found to exhibit altered accumulation in NPB-Piz-t throughout the course of infection. At 0 hpi, three metabolites putatively identified as amino acids or amino acid metabolites showed increased accumulation in *Piz-t*, while eight showed decreased accumulation. By 48 hpi, four metabolites putatively identified as being related to amino acid synthesis showed increased accumulation, while three exhibited decreased accumulation. By 96 hpi, the number of differentially accumulated metabolites tentatively identified as either amino acids or being related to amino acid metabolism increased considerably, with nineteen showing increased accumulation and eight showing decreased accumulation. Notably, features tentatively identified as L-glutamic acid, proline, alanine, and arginine showed increased accumulation in NPB-Piz-t at 96 hpi (Tables S7–S12). When reviewing the metabolites associated with amino acid metabolism, it was also noted that metabolites tentatively identified as being related to arginine metabolism were particularly well-represented across all time points assayed (Tables S7–S12).

Carbohydrate metabolism (including glycolysis/TCA): The importance of carbohydrate metabolism in modulating host susceptibility and the progress of pathogen infection has been well-established [33]. Interestingly, in NPB-Piz-t, a greater number of metabolites related to carbohydrate metabolism exhibited decreased rather than increased accumulation at 0 hpi (nine decreased and one increased). At 48 hpi, fewer differentially accumulated metabolites related to carbohydrate metabolism were observed than at 0 hpi (none increased and three decreased). However, by 96 hpi, a larger number of metabolites tentatively identified as being related to carbohydrate metabolism exhibited differential accumulation in NPB-Piz-t compared to WT (eight increased accumulation; nine decreased accumulation).

Alkaloids: A final class of metabolites showing differential accumulation across multiple time points post inoculation were alkaloids. However, given the diversity of class of molecules and the diversity of structures associated with alkaloids, making tentative identifications of these molecules was exceptionally difficult. Because of this, we focused primarily on making putative identifications primarily at the “class level” for differentially accumulating alkaloids. At 0 hpi, no compounds tentatively identified as alkaloids showed increased accumulation in *Piz-t* vs. WT, while seven showed decreased accumulation. By 48 hpi, four putative alkaloids showed increased accumulation and four decreased accumulation, most of which appeared to be indole alkaloids, a class of compounds previously identified as playing a role in pathogen resistance in rice and barley [34,35,36] At 96 hpi, nine putative alkaloids showed increased accumulation in *Piz-t*, while seven exhibited decreased accumulation. At this time point, a lack of higher-resolution structural data made it impossible to classify types of alkaloids showing increased or decreased accumulation (Tables S11 and S12).

Miscellaneous metabolites: In addition to the larger clusters of metabolites and metabolic pathways identified above, our analyses identified several individual metabolites that showed differential accumulation in NPB-Piz-t vs. NPB at each individual time point assayed post infection (approximately one to five metabolites showing either increased or decreased accumulation at each time point). These compounds included those putatively identified as being involved in aminobenzoate degradation, benzoate degradation, butanoate metabolism, the pentose phosphate pathway, retinol metabolism, benzoic acid family, cyanoamino acid metabolism, taurine and hypotaurine metabolism, and carbon fixation in photosynthetic organisms (Tables S7–S12).

## 3. Discussion

Plant breeders have employed R genes to control plant diseases and increase yields for over a century. Although many R genes and their cognate Avr genes have been cloned, and numerous interacting proteins and signaling components have been characterized over the last three decades, a clear understanding of both how R genes are activated and how their activation triggers a cascade of immune responses to inhibit pathogen invasion is still lacking. In efforts to control the devastating rice blast fungal pathogen, over 100 R genes have been mapped in the rice genome [3]. Among the 38 cloned R genes, only a few have been studied extensively at the molecular level, due to the discovery of their cognate Avr genes in *M. oryzae*. The AvrPiz-t–Piz-t pair shows the best-characterized R gene-mediated resistance at the molecular level in rice, as over a dozen proteins interacting with either AvrPiz-t or Piz-t have been identified in the last two decades [22,24,25]. In this study, we generated two large sets of transcriptomic and metabolomic data from both *NBP-Piz-t* and NBP following blast inoculation. We performed extensive analyses of these datasets to integrate changes in both gene expression and metabolite accumulation, allowing us to investigate the basis of *Piz-t*-mediated resistance at the “pathway” level.

Our transcriptomic analysis identified 15,571 DEGs in NPB-Piz-t and NPB across the five time points monitored following *M. oryzae* infection. In addition to the 9360 DEGs present in both lines, 2791 DEGs were specifically present only in NPB-Piz-t, while 3366 were present only in NPB (Figure 1B). Based on their expression patterns, the 15,571 DEGs can be classified into five groups (Figure 1C,D). In cluster 2, DEGs found only in NPB but not in NPB-Piz-t were highly induced after 48 hpi. In both cluster 3 and 4, DEGs found only in NPB-Piz-t, but not in NPB, were highly induced after 48 hpi. The only difference between cluster 3 and 4 was that the genes in cluster 3 were still highly induced at 120 hpi in NPB-Piz-t, but not in NPB. Overall, there were small differences in the expression patterns of DEGs between NPB-Piz-t and NPB at 0 and 24 hpi; and 48 hpi seemed to be an important time-point for observing changes in transcriptomic expression patterns, with levels of DEGs at this point showing significant divergence between incompatible (NPB-Piz-t) and compatible (NPB) reactions (Figure 1C,D). In the GO term analysis of the DEGs in cluster 2, we found that chitin metabolic processes, chitin catabolism, aminoglycan catabolic processes, aminoglycan metabolic processes, cell wall macromolecule metabolic processes and responses to biotic stimulus are enriched and likely important in response to blast infection (Figure 2A). In the GO term analysis of the DEGs in cluster 3, we found that biological processes involved in mitochondrial development and stress responses are important for blast infection (Figure 2B), as reported previously [26]. In the GO term analysis of the DEGs in cluster 4, we found that following blast infection, DEGs involved in DNA replication and initiation, as well as the phenylpropanoid catabolic and lignin biosynthesis pathways to inhibit fungal invasion, were highly enriched (Figure 2C).

In addition, genes associated with both primary (sugar, fatty acid, amino acid, phenylalanine) and secondary (phenylpropanoid) metabolism showed altered expression across all time points monitored post infection (Figure 3). The patterns of expression of DEGs associated with specific metabolic pathways correlated well with our metabolomic analyses, which identified metabolites from many of these primary and secondary metabolic pathways, as well as associated branches of these pathways, as showing altered accumulation in NPB-Piz-t vs. WT throughout the course of rice blast infection.

The metabolism of fatty acids and several other membrane components (including one sphingolipid) was also significantly altered in NPB-Piz-t compared to WT, following *M. oryzae* infection. This was interesting, as sphingolipid signaling has been implicated in programmed cell death (PCD) and hypersensitive responses (HRs) in plants [32]. However, as our extraction protocol was not optimized for the isolation of sphingolipid membrane components, more study is needed to determine the role of these compounds in pathogen resistance in *Piz-t.* Interestingly, across multiple time points (particularly 0 and 96 hpi) several decanoic acid or decanoic acid derivatives showed decreased accumulation in NPB-Piz-t vs. WT. This was particularly true by 96 hpi, when multiple decanoic acid metabolites showed decreased accumulation in *Piz-t.* It is also possible that by reducing decanoic populations, *Piz-t* populations are depriving the blast pathogen of fatty acid substrates for use in the synthesis of blast membranes.

Both our transcriptomic and metabolomic data indicate that both phenylpropanoid metabolism and downstream flavonoid metabolism are also likely to participate in *Piz-t*-mediated resistance. These data are in agreement with the published work supporting a role for flavonoids in plant disease resistance [27]. In our assays, the levels of the flavonoid metabolites glepiotin B and Angusticornin B were significantly increased in NPB-Piz-t at 0 and 48 hpi after infection. Previous studies have indicated that these two metabolites function as antimicrobial agents [29,30]. As flavonoids serve as ROS quenchers, lower levels of specific flavonoids may result in localized increases in ROS levels during pathogen infection, and contribute to resistance in *Piz-t*.

Both transcriptomic and metabolomic analyses also implicated amino acid metabolism as a component of blast resistance in *Piz-t.* Metabolomic analyses identified several specific amino acid synthesis pathways as exhibiting altered activity in *Piz-t* across the course of infection. For example, metabolites linked to arginine, glycine, cysteine, lysine and tyrosine metabolism showed increased accumulation in *Piz-t* compared to NPB. Notably, many amino acid compounds related to arginine biosynthesis were found at 96 hpi. Multiple studies have shown that, in addition to serving as a key regulatory hub for nitrogen metabolism, arginine and related compounds help balance plant growth between development and defense mechanisms [37].

We present transcriptomic and metabolomic data detailing shifts in gene expression and pathway-level metabolite accumulation in *Piz-t* plants vs. the NPB control line throughout the course of a multi-day *M. oryzae* infection. The combined analyses of gene expression and metabolomic data have highlighted several metabolic pathways (carbohydrate, fatty acid, phenylpropanoid, flavonoid, and terpenoid metabolism) likely to contribute to blast resistance in NPB-Piz-t. Further work is needed, however, to define the role of specific genes and to identify specific metabolites playing a role in modulating this resistance. In particular, targeted metabolomic assays and structural resolution approaches (most likely nuclear magnetic resonance) will be required to confirm the identity of metabolites tentatively identified in this study, and to generate identities for unknown compounds identified here, particularly the complex terpenoid and alkaloid species. As these compounds and their corresponding synthetic genes are confirmed, they can be integrated into metabolic engineering and breeding efforts to increase rice resistance to fungal pathogens.

## 4. Materials and Methods

### 4.1. Plant Material, RNA Isolation and Library Preparation

Two-week-old NPB-Piz-t and NPB leaves were harvested at five time points: 0, 24, 48, 96, and 120 hpi after inoculation with *M. oryzae* isolate RO1-1. The harvested tissues were immediately placed in liquid nitrogen and homogenized with a Qiagen tissue lyser with metal beads for total RNA extraction. Total RNA was extracted using the FastPure Universal Plant Total RNA Isolation Kit (Vazyme, Nanjing, China) following the manufacturer’s protocol for RNA-seq analysis. The purity and quantity of RNA were measured with the NanoDrop 2000 spectrophotometer (Thermo Scientific, Waltham, MA, USA), and RNA integrity was assessed using the Agilent 2100 Bioanalyzer (Agilent Technologies, Santa Clara, CA, USA). The libraries were then constructed using the VAHTS Universal V6 RNA-seq Library Prep Kit according to the manufacturer’s instructions. The transcriptome sequencing and analysis were conducted by OE Biotech Co., Ltd. (Shanghai, China).

### 4.2. RNA Sequencing and Differentially Expressed Gene Analysis

Sequencing of the libraries was carried out on an Illumina Novaseq 6000 platform, producing 150 bp paired-end reads. Initially, raw reads in fastq format were processed using fastp to remove low-quality reads, resulting in clean reads. These clean reads were retained for subsequent analyses and mapped to the reference genome using HISAT2. FPKM (fragments per kilobase of transcript per million mapped reads) values for each gene were calculated, and read counts for each gene were obtained using HTSeq-count. Differential expression analysis was conducted using DESeq2. A *Q* value < 0.05 and fold change > 2 or < 0.5 were set as the thresholds for DEGs.

### 4.3. GO Enrichment Analysis

GO term enrichment analysis was conducted using the agriGO v2.0 (http://systemsbiology.cau.edu.cn/agriGOv2/ (accessed on 1 June 2024) classification system to categorize DEGs into biological processes, molecular functions, and cellular components. Significantly enriched categories were identified based on a hypergeometric test with a Benjamini–Hochberg false discovery rate (FDR) correction. The biological processes term was selected with a fold change ≥2.0 and significance threshold of *p* < 0.05 to identify over-represented GO terms among the DEGs.

### 4.4. KEGG Pathway Analysis

To investigate the functional implications of the DEGs, we performed KEGG pathway enrichment analysis using the R package clusterProfiler (https://github.com/YuLab-SMU/clusterProfiler.git (accessed on 1 June 2024)). The results of the KEGG pathway enrichment were visualized using bar charts. To visualize the connection between multiple biological categories and the list of gene information with expression changes, gene–concept network diagrams were generated using the cnetplot function in the R package clusterProfiler in R Studio (R version 4.3.1). The gene–concept network illustrates the relationship between DEGs and enriched pathways, with node size representing the number of genes involved and edge color indicating fold change in gene expression.

### 4.5. Metabolite Extraction and Sample Preparation

For metabolomic analysis, NPB and NPB-Piz-t plants were grown as described above. Two-week-old rice seedlings were inoculated with *M. oryzae* isolate RO1-1. Leaves were harvested at three different time points, 0, 48 and 96 hpi. All harvested samples were ground in liquid nitrogen using a Qiagen tissue lyser with metal beads. Ground tissues were transferred into a 15 mL conical tube for storage. To extract metabolites, 200 mg of tissue was transferred to a pre-weighed 2 mL microcentrifuge immediately following tissue grinding. Samples were then weighed to the nearest milligram and extraction buffer (methanol containing indole-3-propionic acid at a concentration of 20 µg/mL as an internal standard [ISTD]) was then added to each tube at a ratio of 200 µL per 100 mg tissue (sample masses averaged around 200 mg per sample). Samples tubes were then sealed, and tissues were extracted using gentle nutation at 4 °C for 30 min. Finally, samples were centrifuged at 13,000× *g*, for 15 min, at 4 °C. Supernatants were harvested and passed through a 0.2 µm PVDF disc syringe filter into a low-volume insert seated in an amber LC-MS/MS vial. Biological replicates of each NPB-Piz-t and NPB sample were sent to the Campus Chemical Instrument Center (CCIC) at the Ohio State University for metabolomic analyses.

### 4.6. Metabolomic Analyses and Data Processing

Metabolomic analyses were conducted at CCIC. Untargeted analysis was used on an Agilent 6545 QTOF mass spectrometer with HPLC separation on a Poroshell 120 SB-C18 (2 × 100 mm, 2.7 µm particle size) on an Infinity 1290 LC system. The gradient consisted of solvent A, H2O with 0.1% Formic acid, and solvent B MeOH with 0.1% Formic Acid at a 200 µL/min flow rate with an initial 2% solvent B with a linear ramp to 90% B at 15 min, up to 95% B for 1 min, and back to 2% B at 17 min and an equilibration of 2% B until min 30. Ten µL was injected for each sample and the top 5 ions were selected for data-dependent analysis with a 30 s exclusion window. Mass spectra were recorded at a mass range of 50 to 1700 m/z and a Dual AJS ESI source was used with a VCap of 4000 V, nozzle voltage of 500 V, nebulizer flow of 8 L/min at 250 °C, and sheath flow of 10 L/min at 350 °C. A blank run was conducted every third injection to prevent cross-contamination between sample injections, and blanks were analyzed for sample carry-over.

For feature selection in the untargeted results analysis, including database comparison and statistical processing, samples were initially analyzed in Progenesis QI. Feature alignment scores were all above 90% after peak picking, and normalization was performed using the internal standard of indole-3-propionic acid. ANOVA *p*-value scores between the inoculated NPB-Piz-t and the inoculated NPB samples were calculated, and those features with a *p*-value above 0.05 were removed. With database matching using the PlantCyc and PMN, selecting for adducts M + H, M + Na, M + K, M + 2H and 2M + H and less than 20 ppm mass error, potential metabolites were tentatively identified.

Using the Python program developed by our co-author Shaoxing Dai and the universality of SMILES (Simplified Molecular Input Line Entry System), among numerous databases, SMILES information for each feature was obtained via the putative Chemspider IDs of each peak in the Chemspider database; and additional metabolite information and putative IDs for each feature was acquired from the KEGG and PMN databases using SMILES string searches. Based on these data (i.e., the putative metabolite nomenclature and pathways involved) pathway enrichment analyses were conducted and the abundances of specific metabolites between the inoculated NPB-Piz-t and the inoculated NPB were further investigated. Briefly, employing *p* < 0.05 as the threshold to screen differentially expressed metabolites, the fold change (FC) shift of specific metabolites between the inoculated NPB-Piz-t and the inoculated NPB was calculated using the average normalized peak area for each feature, where FC > 1 indicated increasing expression in the inoculated NPB-Piz-t (up), and FC < 1 indicated decreasing expression (down). Features were then organized by logFC, and sorted in descending order (i.e., metabolites with the biggest increases observed in NPB- Piz-t vs. NPB were listed first). At the three different time points, 0, 48 and 96 hpi, the top 200 metabolites with the largest increase in NPB-Piz-t and NPB at different time points and the top 200 metabolites with the largest decrease in NPB-Piz-t and NPB were then selected and subjected to further analyses. The top 200 up- and down-regulated metabolites were identified manually by screening for the possible metabolites in the KEGG and PMN databases. The putative metabolites were assigned to compound ID(s) for each feature with the highest match score and the lowest mass error. Results of these analyses are provided in Supplementary Tables (S7–S12).

## 5. Conclusions

Overall, this research provides new insights into the transcriptomic and metabolomic changes induced in rice by Avr-R gene interactions during rice blast infection. Analysis of these data sets has allowed us to conclude the following:

1. At several time points post infection, NPB-Piz-t accumulated a range of tentatively identified metabolites with potential antimicrobial activity, including flavonols/flavonoids, glepiotin B, and angusticornin B.
2. Increases in phenylalanine biosynthesis could enhance blast resistance in NPB-Piz-t by enabling increased synthesis of a range of downstream secondary metabolites, including phenylpropanoids, flavonoids, anthocyanins, lignin, tannins and salicylate.

These findings suggest that the *Piz-t* gene mediates resistance through complex changes in both transcriptome and metabolite metabolism, offering potential targets for enhancing rice blast resistance through gene editing and metabolic engineering.

## Data Availability

All data supporting the findings of this study are available from the corresponding author on reasonable request.

## Supplementary Materials

The following supporting information can be downloaded at: https://www.mdpi.com/article/10.3390/plants13233408/s1, Table S1: Combined DEGs through 0, 24, 48, 96 and 120 hpi; Table S2: DEGs in GO analysis of K-means clustering; Table S3: All DEGs in KEGG enrichment pathways of all time points; Table S4: Overlap genes in KEGG enrichment pathways; Table S5: *Piz-t* specific DEGs and *Piz-t* specific GO Term at all time points; Table S6: Metabolites being up-regulated and down-regulated in volcano plots and heat maps; Table S7: Up-regulated metabolites at 0 hpi after RO1-1 inoculation; Table S8: Down-regulated metabolites at 0 hpi after RO1-1 inoculation; Table S9: Up-regulated metabolites at 48 hpi after RO1-1 inoculation; Table S10: Down-regulated metabolites at 48 hpi after RO1-1 inoculation; Table S11: Up-regulated metabolites at 96 hpi after RO1-1 inoculation; Table S12: Down-regulated metabolites at 96 hpi after RO1-1 inoculation.
